# A Robust Fabrication Method for Amphiphilic Janus Particles via Immobilization on Polycarbonate Microspheres

**DOI:** 10.3390/polym10080900

**Published:** 2018-08-10

**Authors:** Karthik Ananth Mani, Noga Yaakov, Yafit Itzhaik Alkotzer, Evgeni Zelikman, Guy Mechrez

**Affiliations:** 1 Department of Food Quality & Safety, Institute for Postharvest and Food Sciences, Volcani Center, ARO, 68 HaMaccabim Road, 7505101 Rishon LeZion, Israel; karthik@agri.gov.il (K.A.M.); nogay@agri.gov.il (N.Y.); yafiti@agri.gov.il (Y.I.A.); 2 Additives and Compounds Division, Tosaf Group, Alon Tavor Industrial Zone, 1812601 Afula, Israel; evgeniz@tosaf.com

**Keywords:** Janus particles, amphiphilicity, polycarbonate, polymethylsilsesquioxane, solvent displacement

## Abstract

Immobilizing particles on beads, fibers, or filaments, when only one side is exposed to the reaction medium and therefore can be selectively functionalized, is a scalable and easy to control strategy for the fabrication of amphiphilic Janus particles. Here we describe a new, robust method for the fabrication of amphiphilic Janus particles based on immobilization of polymethylsilsesquioxane (PMSQ) particles on polycarbonate (PC), a high impact-resistance polymer with superior mechanical properties. The immobilization of the particles on the PC microspores is performed via inverse solvent displacement method. PMSQ particles are added to a PC solution in tetrahydrofuran (THF), a good solvent for PC. The solution is then precipitated by the introduction of aqueous surfactant solution (antisolvent for PC) under an ultrasonic field. It is important to note that THF and water are miscible and do not form emulsion. During precipitation, PMSQ particles are assembled onto the surface of the PC spherical precipitates/microspheres. The exposed hemispheres of the PMSQ particles are then selectively silanized by (3-Aminopropyl)triethoxysilane (APTES) to introduce amine groups on their surface. To increase the polarity of the functionalized hemispheres, the amine groups are further modified to introduce carboxyl groups. SEM characterization confirms the fine embedment of PMSQ particles onto the PC microspheres. Covalent attachment of silica nanoparticles (NPs) to the functionalized hemispheres of the resulting particles along with fluorescent confocal microscopy conclusively prove the successful fabrication of amphiphilic Janus particles. The immobilization of particles onto highly rigid polymeric microspheres such as PC may pave the way for the development of a robust fabrication procedure with high resistance to temperature fluctuations and harsh mixing conditions that can arise during preparation. This method can be implemented toward a large variety of other synthetic commercial polymers such as polyamide, polyether sulfones, Polyether, ether ketone, or similar.

## 1. Introduction

Amphiphilic particles are a special class of materials where the particles are functionalized asymmetrically with domains of hydrophobic and hydrophilic ligands on their respective hemispheres [1,2,3,4,5,6]. Amphiphilic particles are derived from the general case of Janus particles, which were first introduced by the Nobel Prize laureate P.G. de Gennes in 1991 [7]. Named after the Roman god Janus, these anisotropic Janus particles can impart distinctly different chemical or physical properties and directionality within a single particle [4,7,8,9,10]. Janus particles have a number of asymmetrical particle architectures ranging from spherical to different kinds of dumbbell shapes to cylinders and disks [4,11,12,13,14,15]. Their physical properties and self-assembly behavior have been studied in various fields of applications such as particulate surfactants in emulsion stabilization [16,17,18], modulated optical nanoprobes [19,20], and surface catalysts [21,22].

A major challenge is the preparation of multifunctional Janus particles with advanced physical properties. The successful preparation of Janus particles is difficult not only because of the need for increasingly complex and limited synthetic strategies, but also because of experimental characterization of these particles, which is hard to achieve [4,23,24,25]. One dominant approach is the modification of top surfaces of particle monolayers temporarily immobilized on planar surfaces. This can be done by metal deposition [19,26], UV-photopolymerization [27], plasma treatments [28,29], reactive ion etching [30], binding of metal particles [31], or electrochemical growth of metal oxide nanowires [32]. This approach has the advantage of reliability and can be applied to a variety of particle sizes and shapes. Nevertheless, the major drawback is limited interface and complexity of the methods [4,33].

Immobilizing particles on beads, fibers, or filaments, when only one side is exposed to the reaction medium and therefore can be selectively functionalized, is a scalable and easy to control strategy for the fabrication of amphiphilic Janus particles. This approach has been successfully implemented by particle embedment on electro-spun polymer fiber [34,35] and on spherical polystyrene beads [36]. Other advanced methods involve the adsorption of particles to the liquid–liquid interface to form Pickering emulsion of emulsified molten wax and water [37,38,39,40,41].

However in most of the studies the immobilization of particles takes place on soft materials or polymers with relatively low mechanical properties (i.e., low elastic modulus, ultimate strength, and impact resistance) such as wax, polystyrene, and polybutadiene; were the embedment of particles onto a high impact and high strength polymeric cores is challenging. The low mechanical properties of the substrate used for the immobilization might reduce the robustness of the preparation process and limit the scalability of the methods towards large scale operations. Unlike the previous methods, the current study presents the immobilization of particles on a high impact-resistance polymer with superior mechanical properties, such as polycarbonate (PC). The superiority of PC enables to develop a robust fabrication procedure for Janus particles, and to obtain high resistance against temperature fluctuations and against harsh mixing conditions which might arise during preparation.

Yabu et al. implemented methods for precipitation of polymer solutions such as self-organized precipitation and coprecipitation methods to fabricate core-shell structures of nanoparticles (NPs) (shell) immobilized onto Janus polymeric particles (core) [42,43,44,45,46,47,48]. Different types of particles were immobilized by this method such as, Au [45], Fe_3_O_4_ [43,44,46], SiO_2_ [42], and TiO_2_ [43].

**Figure 1:**
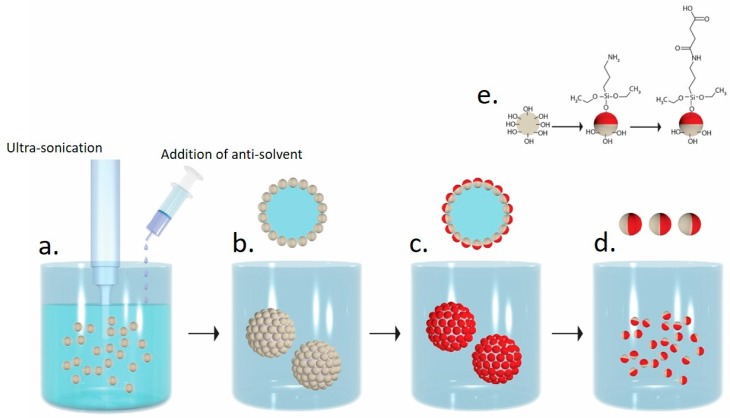
Schematic illustration of the new fabrication method of amphiphilic Janus particles. (**a**) Polymer precipitation by inverse solvent displacement method under an ultrasonic field in the presence of polymethylsilsesquioxane (PMSQ) particles; (**b**) PMSQ particles immobilized onto polycarbonate (PC) microspheres; (**c**) selective silanization of the PMSQ exposed hemispheres by (3-Aminopropyl)triethoxysilane (APTES); (**d**) dissolution of the PC cores for separating the PMSQ amphiphilic Janus particles from the PC; (**e**) schematic illustration of the silanization and the amidation reactions.

This study presents a new, robust, and rapid approach for the fabrication of amphiphilic Janus particles based on the immobilization of polymethylsilsesquioxane (PMSQ) particles onto PC microspheres via inverse solvent displacement method [49,50]. The resulting microspheres are characterized with superior mechanical properties as they are made from polycarbonate. Unlike in the Pickering emulsion approach for fabrication of Janus particles [37], in this study polymer (PC) solution was precipitated via the introduction of antisolvent (water) to the polymer solution; water and tetrahydrofuran (THF) are miscible and do not form emulsion. PMSQ particles were added to a PC solution in THF. Subsequently, the solution was precipitated by the introduction of a dodecylethyldimethylammonium bromide (DDAB) aqueous solution (serves as an antisolvent for PC) under an ultrasonic field (Figure 1a). During precipitation, the PMSQ particles were assembled on the surface of the PC microspheres (Figure 1b). The exposed hemispheres of the PMSQ particles were then selectively silanized by (3-Aminopropyl)triethoxysilane (APTES) such that the embedded hemispheres do not have any contact with the reaction medium and thus are not functionalized (Figure 1c). After silanization, the resulting particles were separated from the PC microspheres by dissolving the PC with THF (Figure 1d). The amine functionalized particles were further modified to form carboxyl functional groups on their surface to increase the hydrophilicity of the functionalized hemisphere (Figure 1d). The silanization and amidation reaction steps are shown in Figure 1e. The high elastic modulus, ultimate strength, and impact resistance of the PC microspheres opens up the potential to scale up the method to an industrial level. The fabrication of PMSQ amphiphilic Janus particles enables to implement these particles toward large verity of applications, due to their good biocompatibility, nontoxicity, and high chemical stability [51].

## 2. Materials and Methods

### 2.1. Materials and Buffers

Polycarbonate (PC, granular, 3 mm nominal size), (3-Aminopropyl)triethoxysilane (APTES), 99%, succinic anhydride, ≥99%, *N-*(3-Dimethylaminopropyl)-*N′-*ethylcarbodiimide hydrochloride (EDC), ≥98%, *N*-*N*-Diisopropylethylamine (EDIPA), 99.5%, MES hydrate, ≥99.5%, dodecylethyldimethylammonium bromide (DDAB), ≥98%, and 6-aminofluorescein, ≥95% were purchased from Sigma-Aldrich (St. Louis, MO, USA). PMSQ (Tospearl 120, dimeter of ~2 µm) were purchased from Momentive, Waterford, NY, USA. Silica nanoparticles (Aerosil OX 50, 40 nm, Evonik, Hanau, Germany). Methanol, ethanol, acetonitrile (ACN), toluene, and tetrahydrofuran (THF) of analytical grade with purity ≥99%, as well as ultra-pure deionized water (ULS/MS grade) were used as received without further purification. 2-(4-Morpholino)ethanesulfonic acid (MES) was purchased from Sigma-Aldrich Chemicals. Buffer 0.05 M was prepared by dissolving the appropriate amount of MES in deionized water.

### 2.2. Preparation of Polycarbonate/Polymethylsilsesquioxane (PC/PMSQ) Microspheres

The inverse solvent displacement method for precipitation of polymer solution was utilized for the fabrication of PMSQ amphiphilic Janus particles. 0.2 g of PMSQ particles were added to 10 wt % PC solution in THF (2 mL). To this mixture, 5 mL aqueous solution (antisolvent) of DDAB (60 mg/L) was slowly added, under an ultrasonic field (20% amplitude, Sonics Vibra-cell ultrasonic liquid processor, Model-VCX 750, Newtown, CT, USA), at a rate of 1 mL/min, resulting in precipitation of micron-scale PC precipitates with adsorbed PMSQ particles on their surface to form PC/PMSQ microspheres. Subsequently, the microspheres were filtered and rinsed with deionized water to remove excess and weakly attached PMSQ particles. The PC/PMSQ microspheres were then dried at 35 °C under vacuum for ca. 3 h.

### 2.3. Fabrication of PMSQ Amphiphilic Janus Particles

#### 2.3.1. Fabrication of PMSQ-NH_2_ Amphiphilic Janus Particles

For the silanization of the APTES molecules (amine edge group) to the exposed hemispheres of the PMSQ particles, 2 mmol of APTES in 10 mL methanol solution were added to the dried PC/PMSQ microspheres and stirred at 500 rpm for 48 h under ambient conditions forming amine groups onto the exposed hemispheres. After silanization, the reactant mixture was centrifuged at 9000 rpm for 10 min at 25 °C; the same conditions for centrifugation were employed in all further experiments. Subsequently, the PC/PMSQ-NH_2_ microspheres were rinsed five times with methanol to remove excess unreacted APTES. The PMSQ-NH_2_ particles were then separated from the PC precipitates by dissolving the PC cores using THF, followed by five successive cycles of centrifugation and rinsing with THF. Two more cycles of centrifugation and rinsing with ethanol were carried out to remove PC, physically attached APTES, and DDAB from the PMSQ-NH_2_ particles. The resulting particles were then dried at 35 °C under vacuum for ca. 3 h.

#### 2.3.2. Fabrication of PMSQ-COOH Amphiphilic Janus Particles

Modification of the exposed hemispheres of the PMSQ-NH_2_ Janus particles to introduce carboxyl groups was done via an amidation reaction of the amine groups with succinic anhydride to form the *N*-[3-(Triethoxysilyl)propyl]succinamidic acid ligand, which contains a carboxyl edge group (PMSQ-COOH). 0.1 g of PMSQ-NH_2_ particles were added to a 2 mL stock solution of 70 mg of succinic anhydride in 10 mL of acetonitrile together with 0.02 mL of EDIPA [52]. The solution was then stirred for 3 h under ambient conditions. The PMSQ-COOH particles were collected by three successive cycles of centrifugation and rinsing with water and acetonitrile. The PMSQ-COOH particles were then dried under vacuum at 35 °C for ca. 3 h.

### 2.4. Fluorescent Labeling of PMSQ-COOH Amphiphilic Janus Particles

Stock solutions of 100 mg of EDC and 1 mg of 6-aminofluorescein dye were prepared separately, each in 10 mL of 0.05 M MES (pH 6.1) buffer. The carboxyl edge groups of the ligands attached to the PMSQ particles reacted with the amine edge groups of the dye in the presence of EDC to form an amide bond. 0.05 g of the PMSQ-COOH amphiphilic Janus particles were added to a 1 mL mixture of 300 µL of the EDC, 100 µL of the dye solution, and 600 µL of the MES buffer. The solution was then mixed by vortex for 1 h at ambient temperature. Subsequently, the mixture was centrifuged and rinsed with MES buffer to remove excess reactants. EDC was used as a cross-linker to chemically attach the PMSQ-COOH amphiphilic Janus particles to the 6-aminofluorescein dye by primarily reacting with the carboxyl groups and producing an amine-reactive O-acylisourea. This intermediate product reacted with the amino groups of the dye to yield an amide bond, releasing fluorescent-labeled PMSQ particles and urea as a byproduct [53]. The removal of excess dye from fluorescent-labeled PMSQ particles was confirmed by a plate reader analysis of the supernatant solution after centrifugation (Supplementary Materials Figure S1). The fluorescent-labeled PMSQ particles were then dispersed again in the MES buffer for analysis by confocal microscopy.

### 2.5. Synthesis of Amine Functionalized Silica Nanoparticles

1 g Silica NPs were dispersed in 40 mL methanol by mechanical mixing. 2 mM of APTES were added slowly to the solution. The reaction was performed at ambient temperature for 45 min. The amine functionalized silica particles were collected by four cycles of centrifugation followed by ethanol rinsing. The NPs were then dried under vacuum at 35 °C for ca. 3 h.

### 2.6. Coupling of Amine Functionalized Silica Nanoparticles(NPs) to the PMSQ-COOH Amphiphilic Janus Particles

Coupling of the amine functionalized silica nanoparticles to the PMSQ-COOH particles was performed by EDC/ Hydroxysuccinimide (NHS) amidation (according to the procedure described in Section 2.4). The amine groups of the silica NPs were reacting with the carboxyl groups of the PMSQ-COOH particles, enabling to characterize the location of the carboxyl groups by direct observation of the coupled silica particles via High resolution scanning electron microscopy (HRSEM). 0.005 g of PMSQ-COOH particles and 0.002 g of amine functionalized silica NPs were added to a 1 mL mixture of 300 µL of the EDC and 700 µL of the MES buffer. The solution was then stirred for 3 h under ambient conditions. The PMSQ-silica Janus particles were separated from the reaction reagents by eight cycles of centrifugation followed by rigorous agitation with both water and acetonitrile to make sure that only covalently attached particles will remain on the surface of the PMSQ-COOH particles. The particles were then dried under vacuum at 35 °C for ca. 3 h.

### 2.7. Characterization of PMSQ Amphiphilic Janus Particles

Electron microscopy. The surface morphology and cross section micrographs of the PC/PMSQ microspheres and of the PMSQ-silica Janus particles were obtained by a scanning electron microscope (SEM) model MIRA3 from TESCAN (Brno-Kohoutovice, Czech Republic) at a 1 & 5 kV accelerating voltage respectively. Energy-dispersive X-ray spectrometry (EDX) elemental analysis was performed on a MIRA3 SEM at 10 kV using an Oxford Instruments analyzer with AZtec software (Concord, MA, USA).

Confocal microscopy. Labeled samples were analyzed by laser scanning confocal microscopy (Olympus FLUOVIEW FV500, Melville, NY, USA) using Argon laser 488 nm wavelength excitation. Fluorescent emission was recorded at wavelengths range of 500–520 nm. Fluorescence and transmitted light differential interference contrast images were collected simultaneously.

FTIR spectroscopy. Attenuated Total Reflection Fourier Transform Infrared Spectroscopy (ATR-FTIR) analysis for monitoring the surface modification on PMSQ was measured by ATR-FTIR with a Tensor 27 FTIR spectrometer (Bruker, Billerica, MA, USA), ATR (Diamond), resolution 8 cm^−1^, scans 64, in the range of 600–4000 cm^−1^.

Plate reader analysis implemented a BioTek Plate reader (Model-Synergy Neo2 Multi-mode reader, Winooski, VT, USA). The excitation and emission wavelengths of the fluorescein dye were 488 nm and 520 nm, respectively.

## 3. Results and Discussion

### 3.1. Preparation of PC/PMSQ Microspheres

The solvent displacement method involves the dissolution of a given polymer in a good solvent and the mixing of the obtained solution with an antisolvent resulting in a spontaneous formation of precipitate particles via polymer precipitation [49,50]. Inverse solvent displacement method was implemented in the current study. In this process, PC is dissolved in THF at a concentration of 10 wt % by mechanical stirring. Subsequently, PMSQ particles with a diameter of ~2 µm are added to the PC solution, which is then precipitated by the addition of a DDAB (60 mg/L) aqueous solution (antisolvent) under an ultrasonic field at a rate of 1 mL/min. During precipitation, PC/PMSQ microspheres are formed.

The resulting properties of the PC/PMSQ microspheres has been optimized in terms of the yield of particles embedment. The embedment yield of the PMSQ particles onto the PC precipitates was characterized by separation of the PC/PMSQ microspheres from the free, not immobilized PMSQ particles via filtration procedure. In addition, the diameter and the uniformity of the particles embedment of the PC/PMSQ microspheres were characterized by SEM. The microsphere diameter maintained above 100 µm at any studied condition (Supplementary Materials Figure S2) ensuring that only the free PMSQ particles were filtered during the separation procedure. The PC and surfactant concentrations along with the duration and the amplitude percentage of the ultrasonic treatment were tuned in order to meet the demands of the desired parameters of particles embedment along with the size and shape of the PC/PMSQ precipitates.

The effect of the PC concentration on the particles embedment yield was studied. The PC concentrations in the solution ranged from 1–10 wt % at a constant PMSQ/PC ratio of 1:1. The PMSQ/PC solutions were precipitated under ultrasonic field at 20% amplitude for 5 min. The ultrasonication parameters were tuned after obtaining the optimal values of the systems composition (PC, PMSQ, and DDAB concentrations). The structure of the resulting PC/PMSQ precipitates at PC concentrations lower than 3 wt % was fibrous. However above 3 wt %, spherical PC/PMSQ precipitates were formed (Supplementary Materials Figure S3). Therefore the PMSQ embedment yield of the resulting spherical PC/PMSQ precipitates (microspheres) were further characterized at PC concentrations higher than 3 wt %. Various PC/PMSQ solutions at different PC concentrations were studied (5, 7, and 10 wt %) each was prepared at three different PC/PMSQ ratios of 1:1, 2:1 and 3:1. The highest embedment yield was obtained at a PC concentration of 10 wt % and PC/PMSQ ratio of 1:1 (Table 1), therefore this system was further investigated in this study.

**Figure 2:**
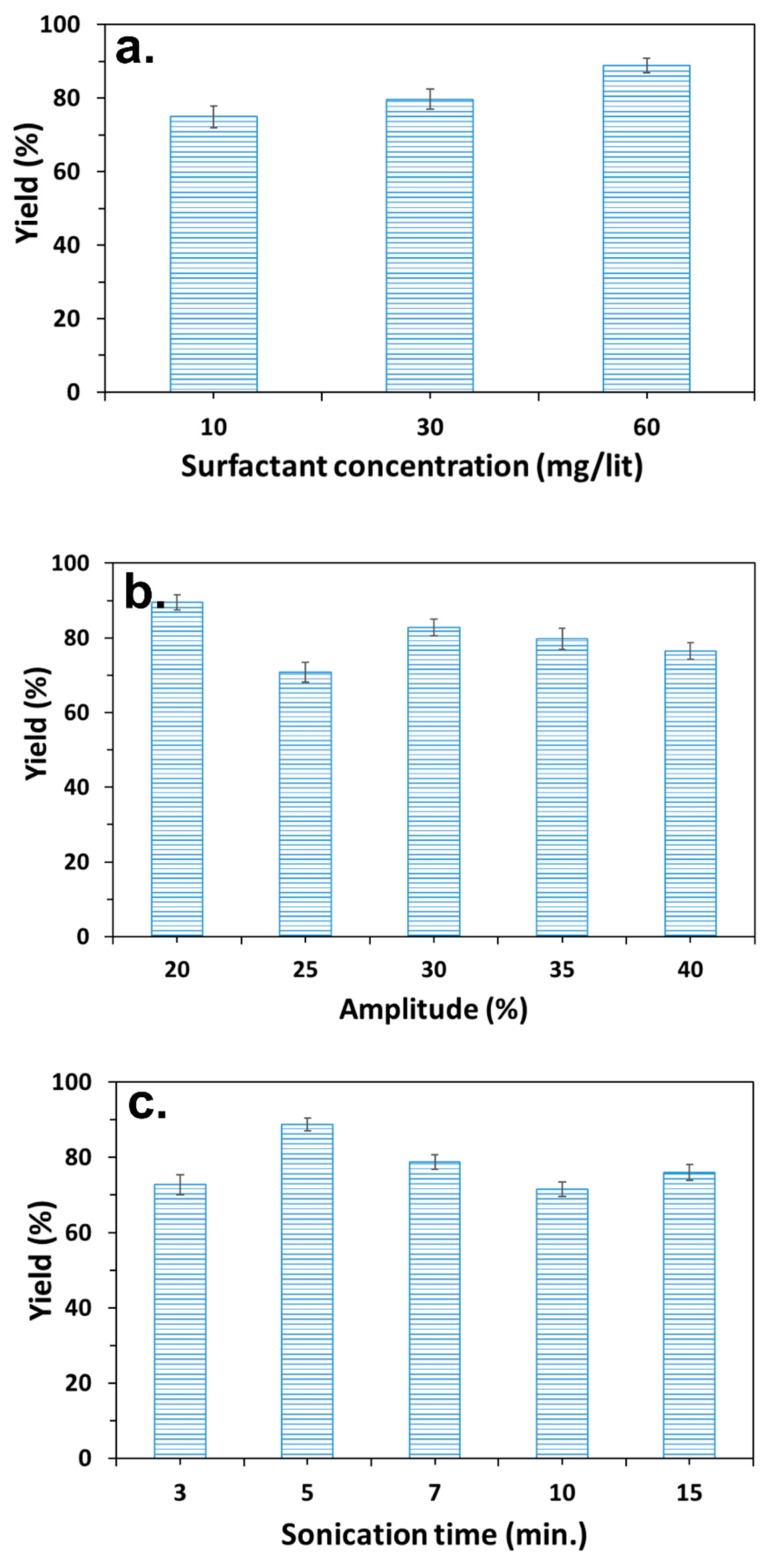
The obtained embedment yield of the PMSQ particles on the surface of PC microspheres vs. the (**a**) surfactant concentration, (**b**) sonication amplitude percentage, and (**c**) sonication time.

The inverse solvent displacement precipitation procedure of the PC/PMSQ systems was performed in the presence of the surfactant DDAB in order to increase the size uniformity of the microspheres [54]. Moreover, it was previously shown that adding surfactants to the system in solvent displacement precipitation method may decrease the agglomeration of the formed microspheres [55]. Precipitation experiments which were conducted in the absence of DDAB have shown significant agglomeration and poor embedment of the PMSQ particles (Supplementary Materials Figure S4). The precipitation experiments of the PC/PMSQ solutions were performed at DDAB concentrations of 10, 30, and 60 mg/L (PC concertation of 10 wt % and PC/PMSQ ratio of 1:1). The highest embedment yield was obtained at the maximum DDAB concentration as can be seen in Figure 2a. Therefore, DDAB concentration of 60 mg/L was chosen as the working parameter for the current study.

The precipitation procedure of the PC/PMSQ solution was performed under ultrasonic field. The ultasonication allows for the obtaining of precipitates with a uniform size and shape. The effect of the ultrasonic treatment parameters on the particles embedment yield and microspheres diameter were characterized. In these experiments the duration of the treatment and the amplitude percentage were varied. The PC and DDAB concentrations along with the PC/PMSQ ratio were fixed in accordance to aforementioned optimal values (10 wt %, 60 mg/L, and 1:1, respectively). The highest embedment yield of the PMSQ particles was obtained at the lowest amplitude percentage of 20% (Figure 2b). The effect of the ultrasonic treatment duration on the particles embedment yield was characterized. The ultrasonication procedure was carried out in four different time intervals of 3, 5, 10, and 15 min. The aforementioned parameters which has led to the highest embedment yields remained fixed in these experiments. Figure 2c shows that a maximum value of the embedment yield is obtained at an ultrasonic treatment with duration of 5 min. The obtained optimal parameters of composition and ultrasonication treatment enabled us to fine-tune the inverse solvent displacement procedure to meet the demands of maximal embedment yield of the PMSQ particles onto the PC microspheres. The shape uniformity and the diameter of the PC/PMSQ microspheres of each studied system was also observed and measured by SEM (Supplementary Materials Figure S2).

**Figure 3:**
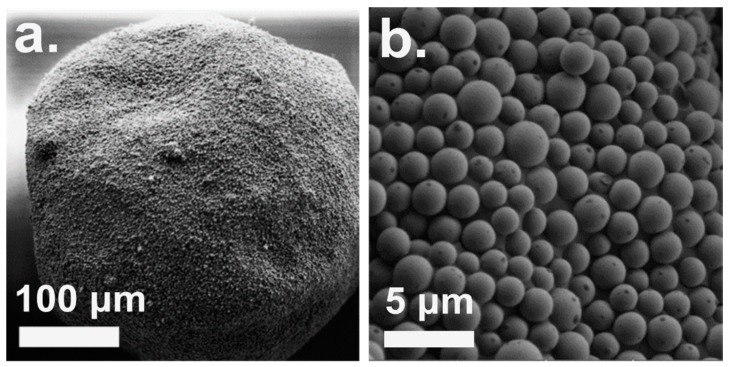
Plan view SEM micrographs of PC/PMSQ microsphere formed via inverse solvent displacement method. (**a**) Overview of a characteristic PC/PMSQ microsphere and (**b**) PMSQ particles embedded on the surface of a PC microsphere.

The structure of the PC/PMSQ microspheres which were fabricated by the above-mentioned optimal parameters was studied using SEM and is depicted in Figure 3. It can be seen that throughout precipitation, the PMSQ particles were assembled on the surface of the PC microspheres in a uniform layer (Figure 3b). The precipitation was performed under an ultrasonic field generating strong hydrodynamic shear-forces in the medium, leading to the formation of PC/PMSQ microspheres with an average diameter of 186 ± 8 µm and a uniform shape compared to standard stirring methods [56]. Some defects in the embedment uniformity are observed, however most of the particles are properly embedded onto the PC microspheres.

**Figure 4:**
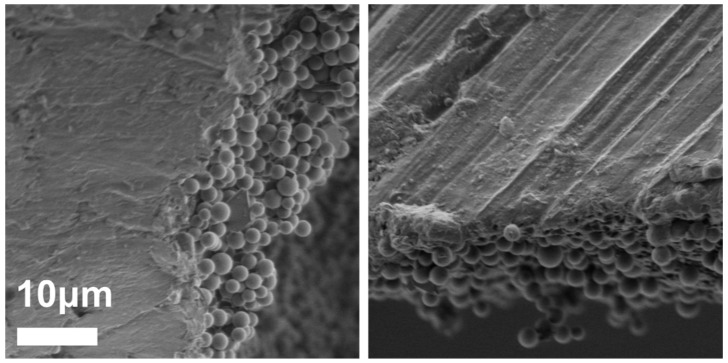
Cross-sectional SEM micrographs of the PC/PMSQ microsphere. It is evident that no PMSQ particles are located inside the PC matrix.

Figure 4 depicts a cross-sectional SEM micrograph of a typical PC/PMSQ microspheres. It can be observed that the PMSQ particles are located solely on the external part of the PC microspheres. The embedment yield of the PMSQ was 89 ± 2%. This finding, together with the fact that the PMSQ particles are assembled in a relatively uniform layer on the surface of the PC core (Figure 2b), are key parameters for achieving a robust method for the fabrication of amphiphilic Janus particles. Moreover, many reported fabrication methods of Janus particles involves mechanical mixing. Shear rates which develop during mixing might lead to structural deformation of the microspheres. This type of deformations can damage the embedment of the particles, which might be highly problematic especially during the stage of the chemical modification (of the exposed hemispheres). Therefore, the structural stability and the rigidity of the microspheres has a great importance. The superior mechanical properties (high elastic modulus, ultimate strength, and impact resistance) of the PC microspheres used in this study leads to a significant structural stability of the resulting PC/PMSQ microspheres against high shear rates during mixing. In addition, unlike in previous studies that show high sensitivity to temperature fluctuations, in the current work, the high thermal stability of polycarbonate allows to obtain high resistance against harsh thermal conditions which might arise during production. Therefore, the current study opens up the possibility to scale-up the new method towards an industrial robust production procedure.

### 3.2. Fabrication of Amphiphilic Janus Particles

The obtained PC/PMSQ microspheres were separated from the THF solution by filtration and rinsed with deionized water. The immobilization of the PMSQ particles on the surface of the PC precipitates serve to selectively functionalize their exposed hemispheres (those facing towards the solution) without any modification of their embedded side, thus forming Janus particles. The PC/PMSQ microspheres were added to an APTES methanol solution to obtain selective functionalization of the exposed PMSQ hemispheres with amine groups via silanization. The PC/PMSQ-NH_2_ microspheres were rigorously rinsed with methanol and centrifuged five times to remove unreacted reagents.

**Figure 5:**
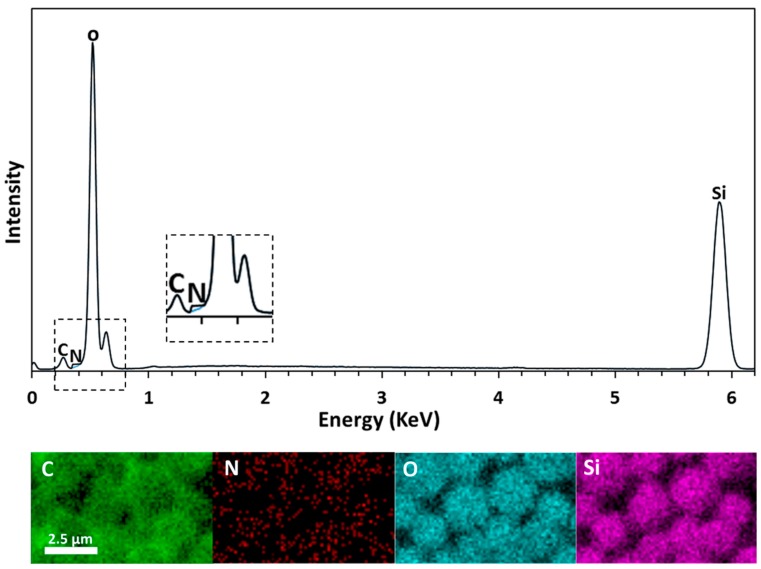
(**Upper**)—EDX curve of PC/PMSQ-NH_2_ (black) and of PC/PMSQ (blue). (**Lower**)—2d map representation of PC/PMSQ-NH_2_ microsphere.

**Figure 6:**
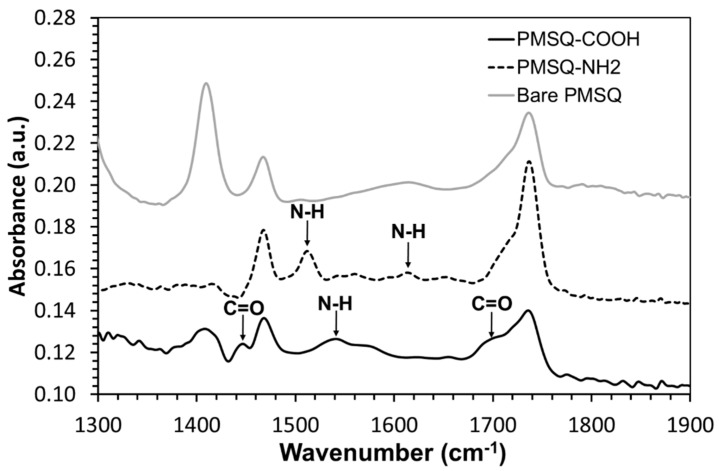
ATR-FTIR spectra of PMSQ-NH_2_ Janus particles (black dashed curve), PMSQ-COOH amphiphilic Janus particles (black curve), and bare PMSQ particles (gray curve).

The PC/PMSQ-NH_2_ microspheres were characterized by EDX spectrometry. The results of the EDX analysis are depicted in Figure 5. The detected elements carbon, nitrogen, oxygen, and silicon are represented in the EDX curve (Figure 5, upper) and in the EDX 2d map representation (Figure 5, lower). The presence of carbon and oxygen is ascribed to both the PC and the PMSQ, but the presence of silicon is ascribed to the PMSQ alone. The low amount of nitrogen is attributed to the APTES molecules (the only component that contains nitrogen in the system), thus supporting the successful covalent attachment of APTES on the PMSQ exposed hemispheres. The rigorous rinsing and the centrifugation cycles after silanization ensured the total removal of physically adsorbed APTES residues such that only covalently bound APTES molecules remained on the surface. In addition, ATR-FTIR spectroscopy was employed to further confirm the successful immobilization of APTES onto the PMSQ particles. The PMSQ-NH_2_ particles exhibited vibration bands of (N–H) bonds at 1511 and 1616 cm^−1^ which are attributed to the presence of an amine group (Figure 6, black dashed curve) [57,58].

The PMSQ-NH_2_ microspheres were separated from the polymeric core through dissolving the PC by THF. The PMSQ-NH_2_ microspheres were then separated from the solution by centrifugation and dried in a vacuum chamber. The PC template can be recycled by rinsing with methanol followed by redissolving the PC in THF and fine filtration to separate the PMSQ residues. To obtain significant amphiphilicity of the Janus particles, their amine groups were further modified to introduce functional groups that are more hydrophilic than the amine groups, such as carboxyl groups. To this end, the PMSQ-NH_2_ Janus particles were re-immersed in a succinic anhydride solution in acetonitrile. The succinic anhydride reacts with the amine groups in the presence of *N*-*N*-Diisopropylethylamine (EDIPA) to form carboxyl groups on the surface of the functionalized PMSQ hemispheres [53]. The embedded hemispheres of the PMSQ particles remain pristine, thus preserving their hydrophobic nature [59].

An ATR-FTIR analysis confirmed the successful introduction of carboxyl groups on the surface of the PMSQ particles (Figure 6, black curve). Clearly, the particles exhibit vibration band of (N–H) at 1540 cm^−1^ and vibration bands of (C=O) at 1446 and 1699 cm^−1^ [52,60]. The existence of these two groups confirms the formation of the amide bond and the presence of carboxyl groups on the surface of the PMSQ-COOH amphiphilic Janus particles. In addition, the bare PMSQ particles do not exhibit these bands (Figure 6, gray curve).

The successful silanization and amidation of the PMSQ particles was confirmed by EDX and ATR-FTIR analysis. In order to prove the amphiphilicity of the particles, we tested whether the functionalization of the PMSQ particles took place solely on their exposed hemispheres. To this end, the carboxyl groups of the PMSQ-COOH particles were labeled by two different approaches: (1) Coupling of amine functionalized silica NPs to the carboxyl groups and characterization of the particles by HRSEM. (2) Chemical modification of the carboxyl groups with 6-aminofluorescein and characterization of the particles by confocal microscopy.

**Figure 7:**
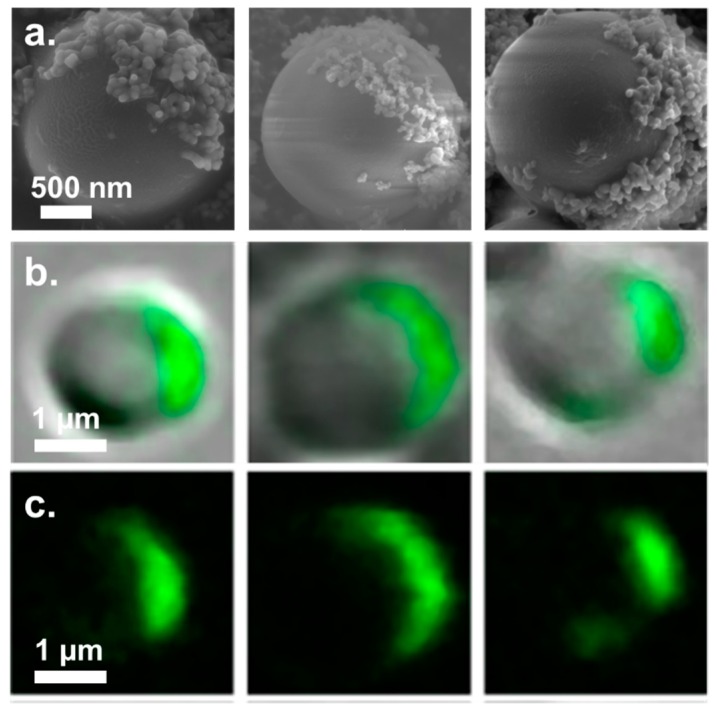
(**a**) HRSEM images of PMSQ-COOH amphiphilic Janus particles decorated with covalently immobilized amine functionalized silica nanoparticles (NPs). The silica-NH_2_ NPs are reacting with the carboxyl groups of the PMSQ-COOH via 1-Ethyl-3-(3-dimethylaminopropyl)carbodiimide (EDC)/*N*-Hydroxysuccinimide (NHS) amidation. (**b**) Overlay confocal microscopy images of the PMSQ-COOH amphiphilic Janus particles labeled with 6-aminofluorescein. (**c**) Fluorescein signal only.

The Janus characteristic of the PMSQ-COOH particles was studied and characterized by covalent attachment of silica-NH_2_ NPs to the functionalized hemispheres of the PMSQ-COOH particles through their carboxyl groups. This procedure allows to label the carboxyl groups of the PMSQ-COOH particles by silica NPs, where their location on the surface of the PMSQ-COOH particles can be easily observed via HRESM. The position of the coupled silica NPs will then reveal the location of the functional groups on the surface of the PMSQ-COOH particles, enabling to directly characterize their Janus property. Yang et al. fabricated micrometer and submicrometer-sized silica Janus particles via the methodology of Garnick et al. In their work the Janus characteristic of the resulting particles was studied by adsorption of gold NPs to their surface [38]. In the current study, we present an improved procedure for characterization of the Janus property based on covalent coupling of the NPs rather than physical adsorption. The NPs are covalently attached to the functional groups which were introduced on the exposed hemisphere (the functionalized hemisphere) which allows us to achieve accurate and direct characterization of the Janus characteristic. Rigorous rinsing of the resulting PMSQ-Silica particles ensures the removal of any silica particles that are physically adsorbed to the surface of the PMSQ particles. The resulting PMSQ-COOH particles, which were labeled by amine functionalized silica NPs (PMSQ-silica particles), were separated and characterized by HRSEM. Figure 7a depicts HRSEM micrographs of characteristic PMSQ-silica particles. The decoration of the silica NPs is observed selectively on a given hemisphere of the PMSQ-COOH particles which clearly confirms the Janus characteristic of the fabricated particles, i.e., during their silanization with APTES while embedded on the PC precipitate, their exposed hemispheres were selectively functionalized. These findings conclusively confirm the successful fabrication of amphiphilic Janus PMSQ particles having a hydrophilic side (containing COOH) and a pristine hydrophobic side [59].

Confocal microscopy images of characteristic PMSQ-COOH particles which where fluorescently labeled through their carboxyl groups are shown in Figure 7b,c. 6-aminofluorescein was reacted with the carboxyl groups of the PMSQ-COOH particles resulting in their selective fluorescent labeling. Figure 7c depicts images that show the fluorescein signal alone. The images further confirms the Janus characteristic of the PMSQ-COOH particles, since unlike the functionalized hemispheres, the embedded hemispheres do not exhibit any fluorescent signal. In order to capture a single amphiphilic Janus particle by confocal microscopy, the particle has to be located in the focal plane at the right orientation. Figure 7b,c depicts characteristic PMSQ particles that were located in the focal plane and therefore their symmetry braking can be clearly observed. This particles were captured in a cross sectional point of view.

The grafting density of the carboxyl groups of the PMSQ particles (the amount of carboxyl groups per unit area) was characterized by labeling of the carboxyl groups with 6-aminofluorescein. The quantification of the number of attached dye molecules allows to directly calculate the number of carboxyl groups introduced on a given PMSQ particle, and then dividing the obtained value by the average surface area of the PMSQ particles is giving the grafting density. The obtained average grating density of the PMSQ particles was 5.444 carboxyl groups/nm^2^ (Supplementary Materials Figure S5).

**Figure 8:**
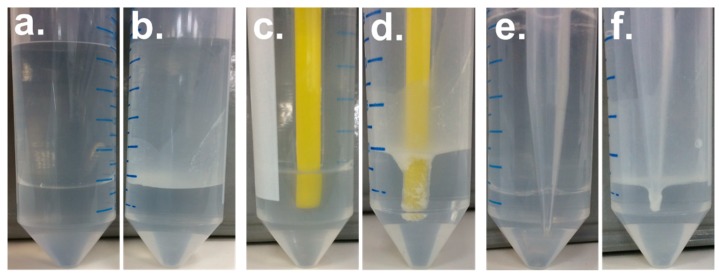
PMSQ-COOH amphiphilic Janus and bare PMSQ particles in a water–chloroform biphasic system. (**a**,**c**,**e**) Bare PMSQ particles. (**b**) PMSQ-COOH amphiphilic Janus particles at the interface. (**d**) PMSQ-COOH amphiphilic Janus particles adsorbed to the polar glass tip caused by the interaction of their hydrophilic hemispheres with the glass surface. (**f**) PMSQ-COOH amphiphilic Janus particles adsorbed to the polypropylene tip as a result of the interaction of their hydrophobic hemispheres with the polypropylene tip.

When introduced to an oil–water biphasic system, studies have shown that amphiphilic particles can self-assemble at the interface. Although these experiments were fairly qualitative, they demonstrated macroscopically the amphiphilic nature of the particles [61,62]. Based on these results, PMSQ-COOH amphiphilic Janus particles were added to a water–chloroform biphasic system. Figure 8 depicts snapshots of bare PMSQ (Figure 8a) and PMSQ-COOH amphiphilic Janus particles (Figure 8b) which were added to a water–chloroform biphasic system. The observed haze at the water–chloroform interface in Figure 8b arises from the self-assembly of the particles, demonstrating their amphiphilic nature. By contrast, the bare PMSQ particles do not self-assemble at the interface, and thus exhibit no haze (Figure 8a). To further pinpoint the amphiphilicity of the PMSQ-COOH particles, a glass tip representing a hydrophilic surface (Figure 8c,d) and a polypropylene tip representing a hydrophobic surface (Figure 8e,f) were each immersed in the biphasic system which contained either the bare PMSQ particles or the PMSQ-COOH amphiphilic Janus particles. In both the glass and polypropylene cases, the particles adsorbed to the surface of the tips (Figure 8d,f). The adsorption of the PMSQ-COOH amphiphilic particles to the polar glass tip is caused by the interaction of their hydrophilic hemispheres (COOH groups) with the glass surface (Figure 8d) and vice versa in the case of the polypropylene tip, where the particles are adsorbed by their hydrophobic hemispheres (untreated/pristine PMSQ) as shown in Figure 8f. No adsorption took place in either case when bare PMSQ particles were added to a water–chloroform biphasic system, as can be seen in Figure 8c,e, respectively. Similar characterization of Janus particles was previously reported in the literature [30].

## 4. Conclusions

This study presents a new robust method for the fabrication of amphiphilic Janus particles by the immobilization of PMSQ particles on PC that contains high elastic modulus, ultimate strength and impact resistance. The PMSQ particles are immobilized on the surface of the PC microspheres via precipitation of PC solution through inverse solvent displacement method. The immobilization of the PMSQ particles on the surface of the PC precipitates made it possible to selectively functionalize their exposed hemispheres without any modification of their embedded side, thus forming amphiphilic Janus particles by introduction of carboxyl groups (highly hydrophilic) on their surface. The Janus characteristics of PMSQ-COOH particles was conclusively confirmed by covalent attachment of silica NPs to their carboxyl functional groups, along with confocal microscopy analysis, both showing that only a single hemisphere of the particles contains the carboxyl groups. Qualitative macroscopic confirmation of successful fabrication of the amphiphilic particles was provided by their self-assembly at the interface of water–chloroform biphasic system.

The immobilization of particles onto a highly rigid polymeric microsphere is challenging, this study presents a method which enables the embedding of PMSQ particles on the surface of polycarbonate microspheres. To the best of our knowledge, this is the first time that an immobilization of particles on a polycarbonate sphere via solvent displacement method is reported. The superior mechanical properties of the PC along with the strong fixation of the PMSQ particles onto the PC microspheres may lead to high resistance against temperature fluctuations, high sheer rates, and other harsh preparation conditions which might arise during production, opening up the possibility to scale-up the new method towards an industrial robust production procedure. Moreover, the ability to recycle the PC template also has an important role in the scale up process. The resulting amphiphilic Janus particles exhibit potential to be implemented in various applications as a new class of colloidal materials with advanced functionalities such as: detection of pathogens, specific cell labelling, in vitro and in vivo imaging, targeted drug delivery [4],lab-on-a-chip devices for the detection of various molecules and development of vehicles for cargo transport [63,64,65], switchable optical elements, reconfigurable materials [39,66,67], and electrode materials for lithium ion batteries [68].

## Figures and Tables

**Table 1:** The embedment yield of the PMSQ particles on the surface of PC microspheres at different PC concentration and PC/PMSQ ratios.

Polymer Concentration (%)	PC/PMSQ Ratio with Yield (%)		
-	**1:1**	**2:1**	**3:1**
1	44.12	-	-
3	43.05	-	-
5	65.49	65.81	51.43
7	78.21	67.46	58.76
10	88.13	82.24	72.03

## Supplementary Materials

The following are available online at http://www.mdpi.com/2073-4360/10/8/900/s1. Figure S1: Fluorescent intensity of the fluorescent-labeled PMSQ particles vs. the number of rinsing cycles. Figure S2: The obtained diameters of the PC/PMSQ microspheres vs. the (a) surfactant concentration, (b) sonication amplitude percentage, and (**c**) sonication time. Figure S3: SEM micrographs of PC/PMSQ microspheres which were prepared at different PC concentrations, (a) 1 wt %, (b) 3 wt %, (c) 5 wt %, (d) 7 wt %, and (e) 10 wt %. Figure S4: Plan view SEM micrographs of PC/PMSQ precipitates in the absence of DDAB. Figure S5: The Intensity of the florescent signal of 6-aminofluorescein as function of its concentration.
