# Supplementary material for: A Robust Fabrication Method for Amphiphilic Janus Particles via Immobilization on Polycarbonate Microspheres
Source: Polymers (Basel). 2018 Aug 10;10(8):900. doi: 10.3390/polym10080900 (PMC6403569; doi:10.3390/polym10080900)
Supplement: Supplementary file 1 [file polymers-10-00900-s001.pdf]

Supplementary Materials:

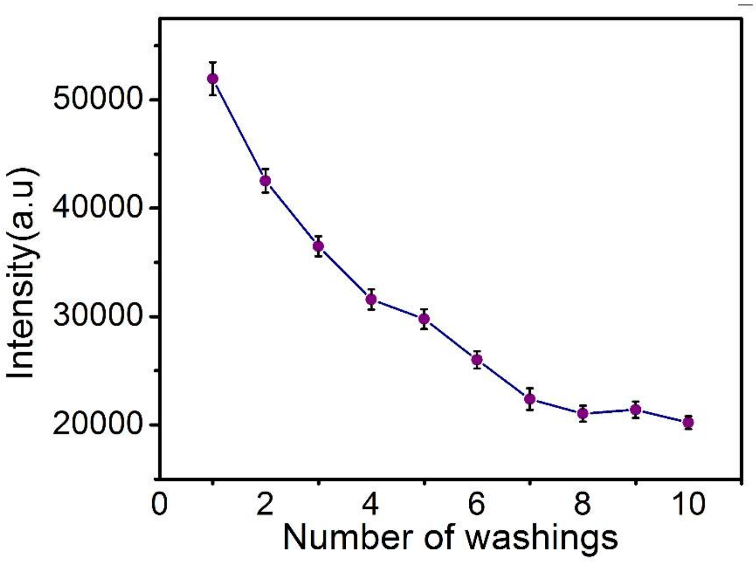

Figure S1. Fluorescent intensity of the fluorescent-labeled PMSQ particles vs. the number of rinsing cycles.

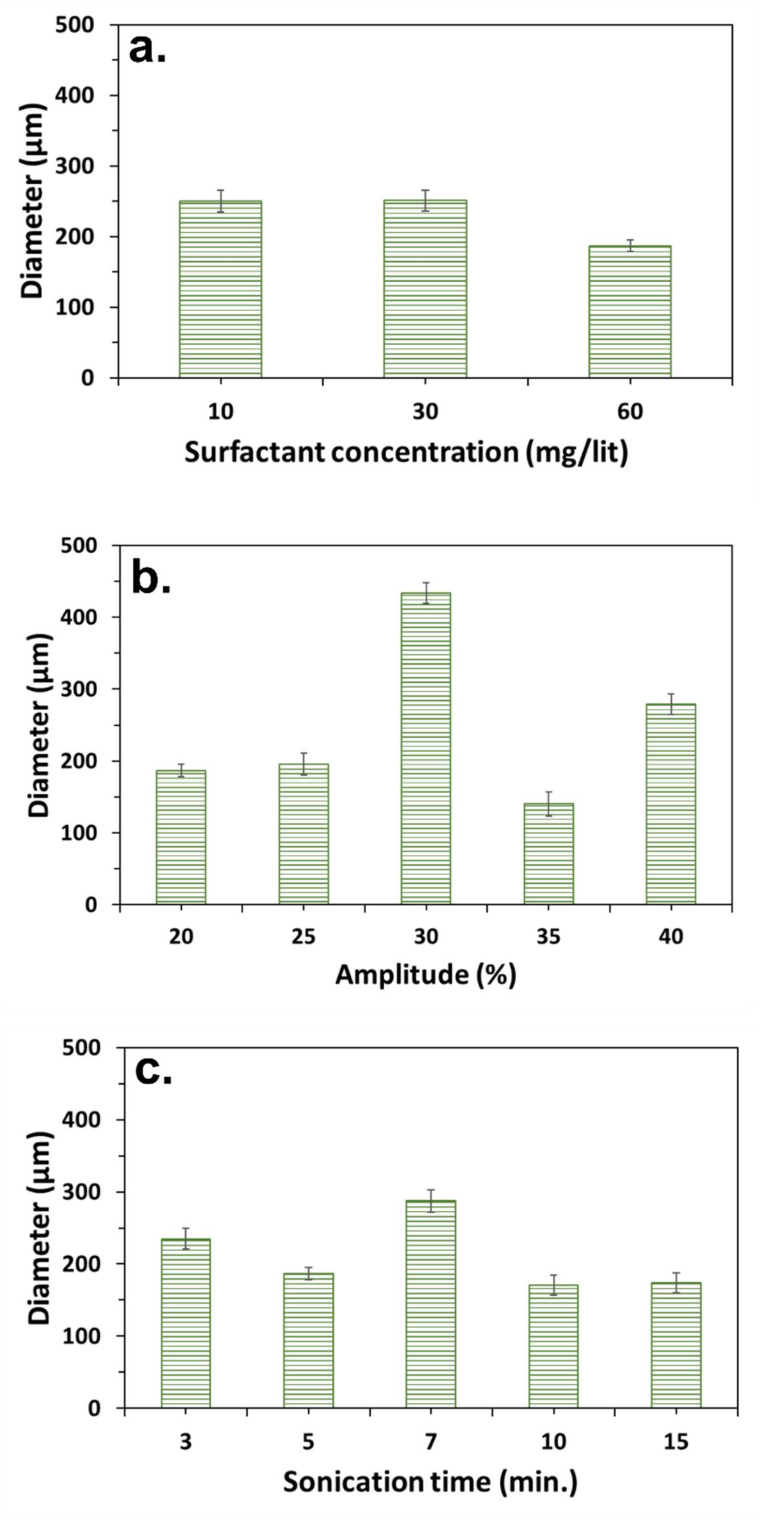

Figure S2. The obtained diameters of the PC/PMSQ microspheres vs. the a) surfactant concentration, b) sonication amplitude percentage, c) sonication time.

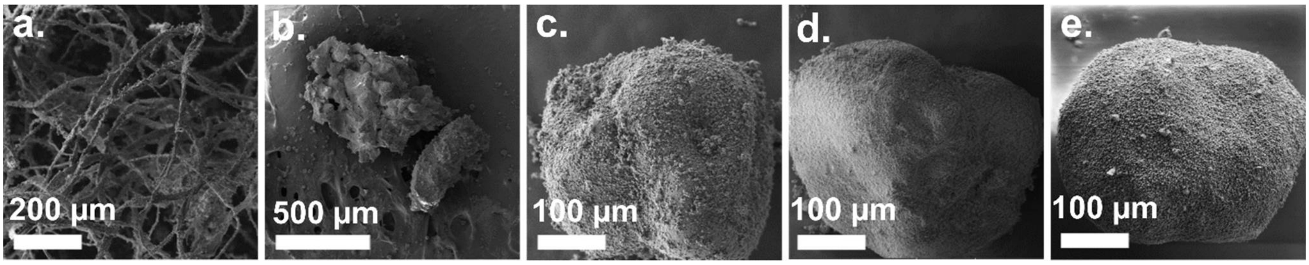

Figure S3. SEM micrographs of PC/PMSQ microspheres which were prepared at different PC concentrations, a) 1 %wt, b) 3 %wt, c) 5 %wt, d) 7 %wt and e) 10 %wt.

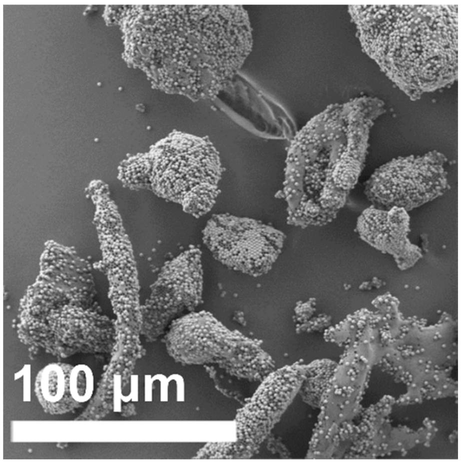

Figure S4. Plan view SEM micrographs of PC/PMSQ precipitates in the absence of DDAB.

Quantification of grafting density for the PMSQ particles

In order to quantify the grafting density of the carboxyl groups of the PMSQ particles (The amount of carboxyl groups per unit area). The particles were labeled by 6-aminofluorescein. First the relationship between the florescent reading and the concentration of 6-aminofluorescein was analyzed. In these experiments, the florescent dye was dissolved in 0.05M MES solution. The florescent readings were measured for different concentrations of 6-aminofluorescein. Linear relationship between the intensity of the fluorescent dye and the concentration of the 6-aminofluorescein was found. The calibration curve is presented in Figure S5.

3.08 mg of PMSQ-COOH particles labeled with 6-aminofluorescein were prepared according to the procedure described in section 2.4. The particles are not Janus particles as the objective of this section is to characterize the grafting density of the functional groups introduced onto the PMSQ particles through the click-chemistry procedures that are implemented in this study.

According to the calibration curve, a control volume of 1ml dispersion (in MES buffer solution) of labeled PMSQ-COOH particles contains 0.022 mg of 6-aminofluorescein attached to their surface (i.e. $6.334X10^{-8}$ moles or $3.814X10^{16}$ molecules of 6-aminofluorescein). The system went through intensive rinsing cycles to remove the un-immobilized dye molecules, thus any remaining 6-aminofluorescein are covalently coupled to the carboxyl groups. The quantification of the number of attached dye molecules allows to directly quantify the number of carboxyl groups introduced on a given PMSQ particle. The following further steps where preformed in order to characterize the grafting density of the studied PMSQ particles:

Calculation of the weight of a single PMSQ particle (the radius, r of the PMSQ particles is 1 μm and their specific gravity, ϱ is 1.23):

$$\text{weight of a single PMSQ particle } = \frac{4}{3}\pi r^3 \cdot \rho \ = 4.189X10^{-12}\ gr$$

Calculation of the number of labeled PMSQ-COOH particles in the control volume of 1ml (in MES buffer solution) dispersion (the total weight of the PMSQ particles was 3.08 mg):

$$total\ number\ of\ PMSQ\ particles = \frac{total\ \text{weight}\ of\ PMSQ\ particles}{\text{weight}\ of a\ single\ PMSQ} = 5.570X10^{8}$$

Calculation of the number of carboxyl functional groups per single PMSQ particle (as mentioned above, the control volume of 1 ml contains total amount of $3.814X10^{16}$ carboxyl groups)

$$number\ of \text{carboxyl}\ groups\ per\ particle = \frac{total\ \text{amount of carboxyl groups}}{total\ number\ of\ PMSQ\ particles} = 6.841X10^{7}$$

Calculation of the grafting density, i.e., the number of functional groups per unit area on a PMSQ particle:

$$number\ of \text{carboxyl}\ groups\ per\ unit\ area = \frac{number\ of \text{carboxyl}\ groups\ per\ particle}{surface\ area\ of\ PMSQ\ particles}$$
$$= 5.444\ carboxyl\ groups/nm^2$$

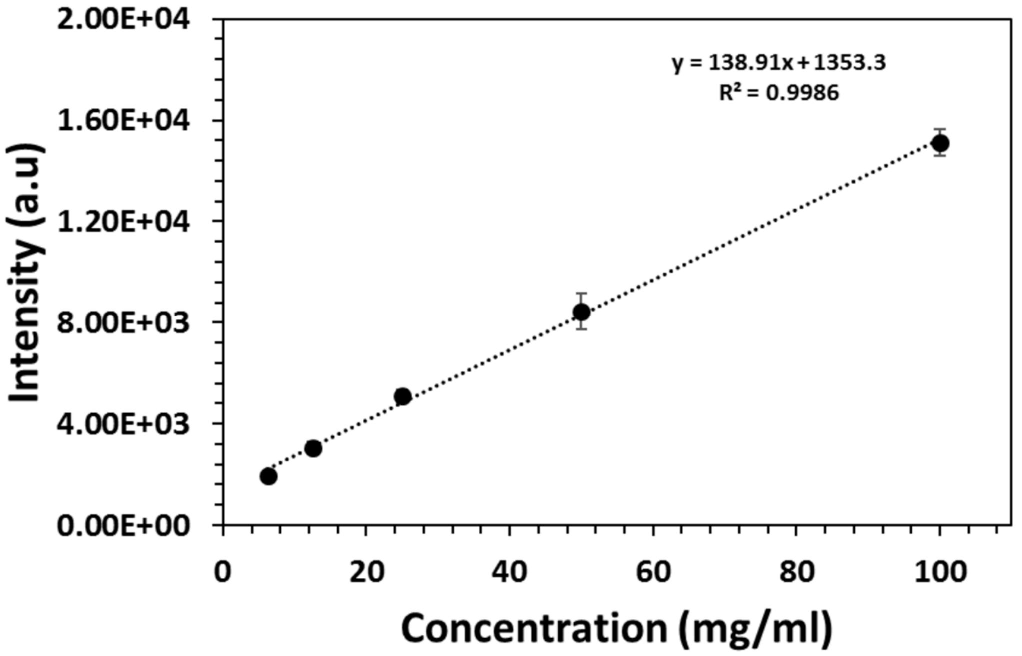

Figure S5: The Intensity of the florescent signal of 6-aminofluorescein as function of its concentration.
